# The Role of PI3K/AKT/mTOR Signaling in Hepatocellular Carcinoma Metabolism

**DOI:** 10.3390/ijms24032652

**Published:** 2023-01-31

**Authors:** Ling-Yu Tian, Daniel J. Smit, Manfred Jücker

**Affiliations:** Institute of Biochemistry and Signal Transduction, University Medical Center Hamburg-Eppendorf, 20246 Hamburg, Germany

**Keywords:** cancer therapy, HCC treatment, glucose metabolism, lipid metabolism, amino acid metabolism, pyrimidine metabolism, oxidative metabolism, tumor microenvironment

## Abstract

Hepatocellular carcinoma (HCC) is one of the leading causes of cancer-related deaths in the world. Metabolic reprogramming is considered a new hallmark of cancer, but it remains unclearly described in HCC. The dysregulation of the PI3K/AKT/mTOR signaling pathway is common in HCC and is, therefore, a topic of further research and the concern of developing a novel target for liver cancer therapy. In this review, we illustrate mechanisms by which this signaling network is accountable for regulating HCC cellular metabolism, including glucose metabolism, lipid metabolism, amino acid metabolism, pyrimidine metabolism, and oxidative metabolism, and summarize the ongoing clinical trials based on the inhibition of the PI3K/AKT/mTOR pathway in HCC.

## 1. Introduction

Primary liver cancer (PLC) is one of the leading causes of cancer mortality worldwide, with around 841,000 new cases and 781,000 deaths every year. About 80% of all PLC are hepatocellular carcinomas (HCC), with increasing incidences over the past few years, making HCC the second leading cause of cancer death in East Asia and the sixth leading cause of cancer death in Western countries [1,2]. The main risk factors of HCC include hepatitis virus infection, auto-immune diseases, drug and non-drug related toxicity, as well as non-alcohol fatty liver disease (NAFLD) [3]. Increasing evidence demonstrates the close connection between metabolic factors, including metabolic syndrome, and the HCC prevalence in NAFLD patients [3]. Metabolic reprogramming is the significant metabolic phenotype of tumors [4,5]. Several metabolic enzymes, involved in glycolysis, lipogenesis, amino acid synthesis, and nucleotide biosynthesis, are augmented to reinforce the anabolic development of tumors in the process of metabolic reprogramming [6]. Malignant tumors can alter their metabolic pathways to sustain the high energy demand for uncontrolled growth and proliferation [7]. The Warburg effect is one of the most observed metabolic changes in malignancy that includes abnormally high glycolysis activity followed by lactate fermentation even in the presence of oxygen [8].

Oncogenic signaling transduction pathways, including the phosphoinositide 3-kinase (PI3K), AKT, and mammalian target of rapamycin (mTOR) pathways, enhance the Warburg effect in tumors, facilitating cancer cell growth and metastasis [9]. The liver plays an important role as a metabolic organ in the maintenance of body metabolic homeostasis and has crucial functions in regulating various metabolic pathways [10]. Cancer with activated PI3K/AKT signaling has been revealed to become more aggressive, and AKT pathway activation has been shown as a notable risk factor for earlier recurrence and poor prognosis in liver cancer patients [11]. Proteins of the PI3K/AKT/mTOR signaling pathway are significantly raised in PET/CT-positive HCC patients, indicating that activation of this pathway might be a key factor of the glycolytic phenotype in HCC cells [12]. Some mTOR inhibitors have been tested for treatment of HCC but have failed in clinical trials, and several new inhibitors on the PI3K/AKT/mTOR pathway are now in clinical trials for HCC patients [13]. However, the knowledge on the interplay of PI3K/AKT/mTOR signaling and its metabolic role in HCC is still limited.

In this review, we highlight the role of the PI3K/AKT/mTOR pathway for the metabolism of HCC, with special emphasis on the molecular interactions. In addition, we offer an update on the ongoing clinical trials targeting the PI3K/AKT/mTOR pathway for HCC therapy.

## 2. The PI3K/AKT/mTOR Pathway in HCC

The PI3K/AKT/mTOR signaling pathway regulates crucial cellular processes in the physiological setting as well as most hallmarks of cancer, including cell cycle, survival, metabolism, motility, and angiogenesis [14]. Dysregulation of the phosphatidylinositol 3-kinase (PI3K) is one of the most frequent events in tumorigenesis [15]. Currently, three classes of PI3K are known in the human genome, i.e., class I, class II, and class III [15]. The Class I PI3K are mainly known to drive tumorigenesis, and the activated Class I PI3K phosphorylates the phosphatidylinositol 4,5- biphosphate (PIP2) to phosphatidylinositol 3,4,5-triphosphate (PIP3) [16]. The Class I PI3Ks include four catalytic units encoded by *PIK3CA*, *PIK3CB*, *PIK3CG*, and *PIK3CD* [17]. In general, mutation of PI3K catalytic isoform p110α is the most common in human cancers, while the catalytic isoforms p110β, p110δ, and p110γ are rarely mutated but can be overexpressed in cancer [18].

PI3K is especially highly expressed in HCC tumor tissue, and the upregulation of *PIK3CA* was associated with HCC proliferation and negatively correlated with apoptosis. In addition, high expression of *PIK3CA* was associated with an unfavorable prognosis in HCC patients [19]. HCC patients with an early-stage recurrence have a higher mutation rate of *PIK3CB* [20]. Previous research demonstrated that certain microRNAs, including miR-142-3p, repress HCC progression and increase apoptosis to inhibit HCC by decreasing the *PIK3CG*-mediated activation of the PI3K/AKT pathway [21]. Regarding MiR-7, which targets *PIK3C*, it has been demonstrated that it controls cell proliferation and metastasis through the PI3K/AKT/mTOR pathway in HCC [22].

There are three Class II PI3K isoforms (PI3K-C2α, PI3K-C2β, and PI3K-C2γ) with different roles. PI3K-C2α plays a prominent role in endocytosis, vesicular trafficking, and mitosis; PI3K-C2β is related to cell migration and mTOR signaling repression and PI3K-C2γ regulates AKT2 activation and glycogen storage [23]. Downregulation of PI3KC2α leads to degraded vascular endothelial growth factor A (VEGFA)-mediated signaling and decreased angiogenesis in human HCC cells [24]. PI3K-C2β plays a crucial role in hepatitis C virus (HCV) propagation in human hepatocellular carcinoma cells [25].

Class III PI3K (i.e., PIK3C3) plays an essential role in cellular processes [26]. Inhibition of PIK3C3 blocks the activation of SGK3, which is the cancer stem cell (CSCs) promoter, and AMP-activated kinase (AMPK), thereby repressing the growth of HCC CSCs in mice [27]. The serine and threonine kinase AKT family includes three isoforms (i.e., AKT1, AKT2, and AKT3). A wide range of diseases, including cancer, is caused by AKT dysregulation [28]. In hepatocytes, expression of AKT1 as well as AKT2, but not AKT3, can be detected [29]. It has been demonstrated that c-MYC activation is strongly correlated with phosphorylated AKT1 expression and that HCC patients with relatively higher expression of AKT1, but not AKT2, have an unfavorable outcome [30]. However, previous research reported that AKT2 impacts the prognosis of HCC patients and that AKT2 may promote cell proliferation and invasion [31]. Furthermore, Galicia et al. reported that PI3K/AKT signaling is activated by loss of the tumor suppressor phosphatase and tensin homolog (PTEN) [32]. The preliminary role of AKT2 in the context of tumor transformation is not for the pro-survival or pro-growth of tumor cells; however, it displays the crucial function of metabolic regulation in the HCC model of mice [32].

The mammalian target of rapamycin (mTOR) kinase is a proficient regulator of protein synthesis that connects nutrient sensing to cell growth and is frequently observed in cancer [33]. The different proteins binding to mTOR activate their function by forming two complexes, which are called mTOR complex 1 (mTORC1) and mTOR complex 2 (mTORC2) [34]. Activation of PI3K and AKT leads to mTORC1 activation and phosphorylation of ribosomal protein S6 kinase 1 (S6K1) and eukaryotic translation initiation factor 4E-binding protein-1 (4E-BP1). The mTORC2 complex includes the rapamycin-insensitive companion of mTOR (Rictor) and phosphorylates AKT, promoting AKT kinase activity [35]. Constitutive activation of PI3K/AKT/mTOR has been reported in cancer [36]. In 50% of HCC cases, an upregulation of the mTOR pathway has been reported, which underlines the role of mTORC1 as a potential therapeutic target [37]. However, the long-term usage of the inhibitors of mTORC1 increases interleukin-6 (IL-6) production, activates the signal transducer and activator of transcription 3 (STAT3), and facilitates HCC development in a murine obesity liver model [38]. For mTORC2, it has been pointed out that hepatic mTORC2 facilitates hepatosteatosis and cancer via de novo fatty acid and lipid synthesis in HCC [39].

## 3. The Role of Metabolic Pathways in HCC

### 3.1. Glucose Metabolism in HCC

Increased aerobic glycolysis is a crucial hallmark of cancer metabolism [40]. Inhibition of the AKT/mTOR signaling pathway decreases the aerobic glycolysis in HCC cells, thereby eventually abolishing their cell growth [2]. It has been reported that liver cancer is often driven by the activation of AKT/mTOR signaling and that glycolysis activity is increased during HCC growth. Therefore, the suppression of AKT/mTORC1 might be a suitable strategy to prevent HCC development [41]. However, mitochondrial glucose oxidation that occurred in HCC cell metabolism independently of the PI3K/AKT/mTOR pathway has also been revealed previously [42]. In HCC cell lines, the Warburg effect is elevated with increasing glucose uptake, and it is revealed that miR-873 activates the key glycolytic proteins AKT/mTOR via targeting Nedd4 family-interacting protein 1 (NDFIP1), which initiates metabolic change and causes hepatocellular carcinoma formation and metastasis [43]. Laminin subunit gamma 1 (Lamc 1) decreased the growth of HCC cells by promoting tumor cell death and decreased glucose transportation via the inhibition of expression of pyruvate kinase M2 (PKM2), mechanically reducing the expression of glucose transporter 1 (GLUT1) and lactate dehydrogenase A (LDHA), which implied that the AKT pathway plays a crucial role in the progression of HCC by transforming glucose metabolism [44]. Furthermore, the mTOR kinase is an essential downstream effector of AKT, and activation of mTOR hampers its downstream effector, eIF4E binding protein (4EBP1). This is stimulating the initiation of protein translation, thereby resulting in increased glucose transporter 1 (GLUT1) translocation and hexokinase 2 (HK2) activity, ultimately enhancing glucose uptake and glycolysis [45]. Cui et al. found that inhibitors of AKT and mTOR repressed the motility of liver cancer cells, decreased glucose consumption and lactate production, and hindered HK2 expression, suggesting that inhibition of the AKT/mTOR signaling axis deterred cell motility by repressing glycolysis in HCC cells [46]. Moreover, lower AKT activity results in cell cycle arrest and decreased metabolic flux to glycolysis and the tricarboxylic acid (TCA) cycle to repress tumor growth [47].

**Figure 1:**
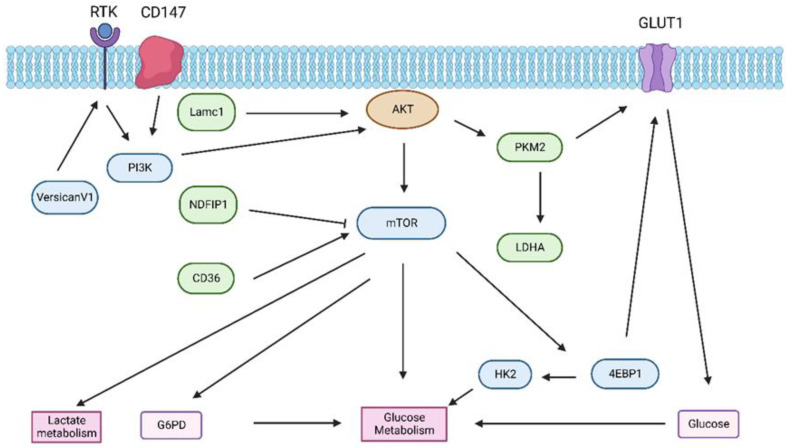
Overview of interaction between the PI3K/AKT/mTOR pathway and glucose metabolism in HCC. Lamc1, laminin subunit gamma 1; NDFIP1, Nedd4 family-interacting protein 1; G6PD, glucose 6 phosphate dehydrogenase; PKM2, pyruvate kinase M2; LDHA, lactate dehydrogenase; HK2, hexokinase 2; GLUT1, glucose transporter 1; 4EBP1, eIF4E binding protein.

Glucose is catabolized by two parallel metabolic pathways, i.e., glycolysis and the pentose phosphate pathway (PPP) [48]. Glucose flux through the glycolytic pathway can be shifted to the pentose phosphate pathway (PPP) [49]. The PPP (also known as the phosphogluconate pathway or the hexose monophosphate shunt) branched from glycolysis at the first step of glucose-6-phosphate (G-6-P). The PPP plays an important role in cancer cell proliferation and growth. 6-phosphogluconate dehydrogenase (6PGD), a key enzyme within the PPP, has been demonstrated to contribute to oncogenesis [50]. Activation of the PI3K/AKT signaling pathway leads to higher G6PD activity, thereby enhancing metabolic activities and promoting cancer cell growth [51]. G6PD overexpression promotes growth in normal liver cells, whereas targeting G6PD decreases HCC growth and HCC cell survival [52]. Cheng et al. reported that there was an essential reciprocal crosstalk between the PI3K/AKT signal and PPP metabolic pathways [53]. PI3K/AKT activation stabilizes G6PD, the rate-limiting enzyme of the PPP, by inhibiting the E3 ligase tripartite motif-containing protein 21 (TIRM21) and promotes the PPP, which in turn supports AKT activation and additionally enables cancer metabolic reprogramming by suppressing the expression of the AKT inactivator pleckstrin homology-like domain family A member 3 (PHLDA3) [53]. VersicanV1, which is the protein of the extracellular matrix, could promote the Warburg effect of HCC cells through the epidermal growth factor receptor (EGFR)–PI3K–AKT signaling pathway, consequently increasing the proliferation, invasion, and metastasis of HCC cells [54]. Furthermore, cluster of differentiation 36 (CD36), an integral transmembrane glycoprotein, prompted mTOR-mediated oncogenic glycolysis via activation of the PI3K/AKT signaling axis in HCC [55] (Figure 1).

Lactate accumulation in cancer—a hallmark of the Warburg effect—has recently been demonstrated to control cancer cell metabolism and survival [56]. Kirk et al. have reported that CD147 plays a pivotal role in lactate transport, indicating that CD147 initiates the activation of the PI3K/AKT signaling axis, hence controlling lactate export in liver cancer cells [57] (Figure 1). A prior investigation has also described a correlation between lactic acidosis and activity of the PI3K/AKT pathway in cancer cells [58] (Figure 1).

### 3.2. Lipid Metabolism in HCC

**Figure 2:**
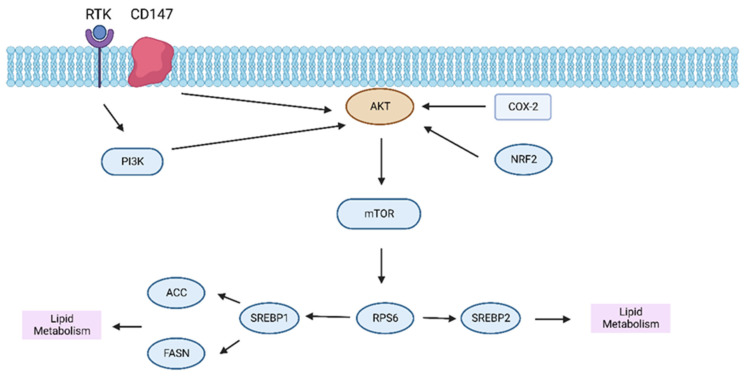
Interplay between the PI3K/AKT/mTOR signaling and lipid metabolism in HCC. Abbreviation: ACC, acetyl-CoA-carboxylase; FASN, fatty acid synthase; SREBP1, sterol regulatory-element binding proteins 1; RPS6, ribosomal protein S6; COX-2, cyclooxygenase 2; NRF2, nuclear factor erythroid 2-related factor 2; SREBP2, sterol regulatory-element binding proteins 2.

Lipid metabolism has been implicated in tumorigenesis as an essential energy supplier, sustaining cell growth, and delivering the intermediate substances for biosynthesis in the cancer cells [59]. It has been previously reported that fatty acids and cholesterol are crucial for the growth and progression of tumors, as they are the elements of the cell membrane [60]. The liver is an essential organ for energy metabolism, lipid biogenesis, as well as the distribution of lipids [61]. An anomalous lipid metabolism was observed in liver cancer cells generated by hydrodynamic injection of activated forms of AKT and Nras via a transposon system into mouse hepatocytes [62]. The activation of the AKT/mTOR signaling pathway downstream of transmembrane glycoprotein CD147 triggers the transcription of key fatty acid-related genes, including the fatty acid synthase and acetyl-CoA-carboxylase (ACC), to facilitate the fatty acid synthesis in HCC cells [63]. The AKT/mTOR/SREBP-1 (Sterol regulatory-element binding protein 1) signaling pathway is a key pathway to regulate hepatic cellular lipid metabolism [64]. In HCC cell lines, the inhibition of AKT targets lipogenesis in the HepG2 cancer cell line [65]. Sterol regulatory-element binding proteins (SREBPs) are transcription factors that regulate the expression of genes involved in lipid synthesis [66]. Furthermore, the AKT/mTORC1/S6 pathway promoted lipogenesis via transcriptional and post-transcriptional mechanisms that include inhibition of fatty acid synthase ubiquitination by the USP2a de-ubiquitinase and disruption of the SREBP1 and SREBP2 degradation complexes [67]. Furthermore, suppression of the adenosine triphosphate citrate lyase, acetyl-CoA carboxylase, fatty acid synthase, stearoyl-CoA desaturase 1, or sterol regulatory element-binding protein 1, which are involved in lipogenesis, reduced proliferation, and survival of HCC cell lines, and AKT-dependent cell proliferation [67]. AKT/mTOR/SREBP signaling by insulin and growth factors is the preliminary axis in anabolic metabolism, which assembles substances related to the tumor [66]. In addition, it was shown that activation of the AKT/mTOR signaling pathway upregulates SREBP1 expression, which enhances lipid metabolism by activating gene transcription of lipogenesis, thereby promoting the growth of HCC cells [68]. The fatty acid synthase (FASN) is essential for AKT-mediated carcinogenesis of HCC [69]. While the dependency on the FASN was demonstrated in HCC, it could not be shown for cholangiocarcinoma [70]. Celecoxib, a non-steroidal anti-inflammatory drug, targets the cyclooxygenase 2 (COX-2)/AKT pathway and was sufficient to inhibit the progress of HCC by inhibiting lipogenesis [71]. Within the hypoxic tumor microenvironment, the activated AKT/mTOR pathway causes lipogenesis and lipid accumulation during HCC progression and leads to proliferation, increased viability, and angiogenesis [72]. In non-transformed and non-proliferative hepatocytes, nuclear factor erythroid 2-related factor 2 (NRF2) directly triggers transcription of growth factor genes encoding the platelet-derived growth factor receptors ligand platelet-derived growth factor C and the EGFR ligands transforming growth factor alpha and amphiregulin, which contribute to AKT activation via autocrine signaling to control hepatic glucose and lipid metabolism [73] (Figure 2).

### 3.3. Amino Acid Metabolism in HCC

**Figure 3:**
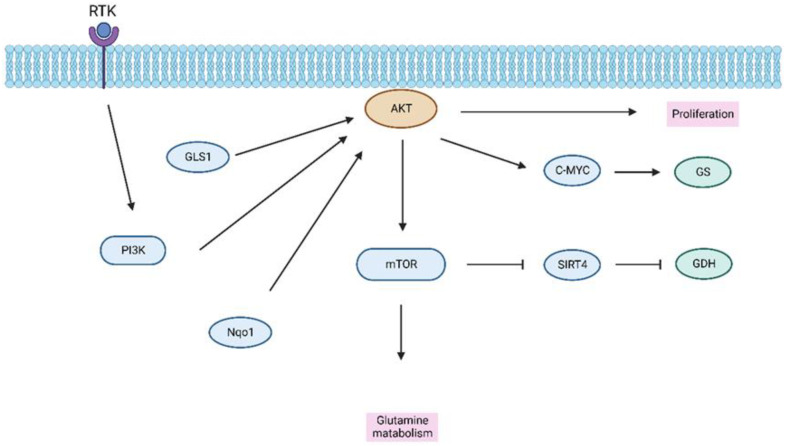
Regulation of the glutamine metabolism of the PI3K/AKT/mTOR pathway. Abbreviation: GLS1, Glutaminase 1; Nqo1, NAD(P)H quinone dehydrogenase 1; SIRT4, Sirtuin 4; GS, Glutamine synthetase; GDH, Glutamine dehydrogenase.

Glutamine is one of the most common amino acids [74]. Rapidly proliferating cells, such as immune cells, as well as cancer cells, have a high demand for glutamine [75]. Cancer cells increase their rate of glucose and glutamine metabolism for bioenergetic and anabolic intents, in which a substantial amount of external carbon sources are redirected to produce DNA, proteins, and lipids that are required for proliferation [76]. Activation of PI3K/AKT signaling leads to glutamine utilization in the metabolic pathways to promote proliferation [51]. Glutamine can activate mTORC1 through the mTOR signal pathway by Rag GTPase-dependent and -independent mechanisms [77]. Liver cancer bears a metabolic dependency on glutamine, and exploiting metabolic vulnerabilities may be a promising target for the treatment of liver cancer [78]. The glutaminase 1 (GLS1), the key enzyme in glutamine metabolism, is highly expressed in HCC tissue compared to healthy liver tissue. Furthermore, GLS1 was previously correlated with clinicopathological features and a poorer prognosis in HCC patients. The potential mechanism is that GLS1 activates the AKT pathway to promote the proliferation of HCC [79]. Hepatocellular carcinoma tumorigenesis is facilitated by glutamine metabolism through the activation of a positive feedback loop involving the mTORC2/AKT/C-MYC axis. Subsequent upregulation of glutamine synthetase (GS) expression and mTORC1 signaling ultimately release the influence of sirtuin4 (SIRT4) on glutamate dehydrogenase (GDH) [80]. N-Myc downstream-regulated gene 2(NDRG2)-dependent inhibition of c-Myc via the PI3K/AKT pathway can further suppress glutaminolysis in tumor cells [81]. Amino acid-deficient diets and disruption of arginine metabolism have been shown to be a potential nutritive treatment for tumor patients, including HCC patients [82,83]. The previous results from Saha et al. showed that tumors with basal PI3K/AKT activity should be sensitive to amino acid deprivation; however, those with high PI3K/AKT pathway activity should be resistant. Amino acid deprivation could still induce quiescence in normal cells, accordingly setting up the proliferating cancer cells to be more sensitively affected by drugs that target tumor cells [83,84]. The use of glutamine is important; however, the higher glutamine concentrations may also cause the formation of other metabolic substances, including amino acids, which help the liver to grow [85]. Furthermore, NAD(P)H quinone dehydrogenase 1 (Nqo1) ablation-initiated suppression of the PI3K/AKT pathway, repressed the expression of glutaminolysis-related genes, and drove metabolic adaptation in hepatocytes. Contrarily, *Nqo1* overexpression induced hyperactivation of the PI3K/AKT signaling axis and increased the metabolic adaptation rate [86] (Figure 3). Interestingly, based on the metabolism-related gene used to stratify the HCC patients, the patients in the group with the PI3K/AKT/mTOR signaling pathway show the activation of asparagine synthetase (ASNS), glycolysis, and the pentose phosphate pathway [87].

### 3.4. Pyrimidine Metabolism in HCC

In addition to protein and lipid synthesis, pyrimidine synthesis represents another major anabolic process that is responsible for cellular growth regulation [88]. The nucleotide metabolism is an essential metabolic process that creates purine and pyrimidine for cell proliferation, and a raised nucleotide metabolism sustains the disordered growth of tumors, which is a hallmark of cancer [89].

**Figure 4:**
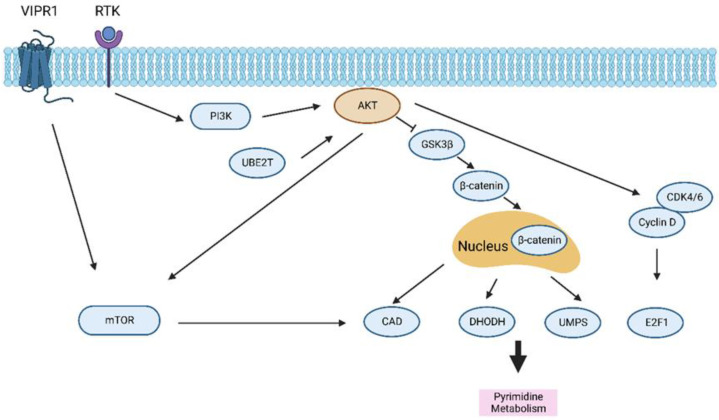
The role of PI3K/AKT/mTOR signaling in regulating pyrimidine metabolism. Abbreviation: VIPR1, vasoactive intestinal polypeptide type-I receptor; UBE2T, ubiquitin conjunction enzyme E2T; CAD, carbamoyl-phosphate synthetase 2, aspartate transcarbamoylase, dihydroorotase; DHODH, dihydroorotate dehydrogenase; UMPS, uridine 5′-monophosphate synthase; E2F1, E2F transcription factor 1; GSK3β, glycogen synthase kinase 3 beta; CDK4/6, cyclin-dependent kinase 4/6.

Growing evidence shows that pyrimidine metabolism can increase tumor malignancy in cells [90]. The expression of key enzymes of the pyrimidine metabolism can predict the prognosis of HCC patients [91]. The ubiquitin-conjugating enzyme E2T (UBE2T) increases the expression of the de novo synthesis of pyrimidine metabolism associated enzymes, including carbamoyl-phosphate synthetase 2, aspartate transcarbamoylase, dihydroorotase (CAD), dihydroorotate dehydrogenase (DHODH), and uridine 5′-monophosphate synthase (UMPS), and enhances the pyrimidine metabolism by facilitating AKT ubiquitination and AKT/β-catenin signaling, thereby promoting the HCC progression [92]. Increased purinergic signaling regulated by PI3K pathway-dependent activation of E2F transcription factor 1 (E2F1) mediated by the cyclin D-cyclin-dependent kinase 4/6 complex facilitates HCC tumorigenesis, indicating the possibility of targeting purine metabolic reprogramming as a potential treatment for patients with HCC [93]. Fu et al. have indicated that the vasoactive intestinal polypeptide type-I receptor (VIPR1) is decreased in HCC and that VIPR1 inhibits HCC growth by regulating the phosphorylation of the mTOR pathway as well as pyrimidine biosynthesis [94]. Moreover, mTORC1 plays a key role in controlling cell biosynthesis and growth, and mTOC1 activation following PI3K/AKT signaling results in the S6K1-mediated phosphorylation of CAD. CAD, in turn, facilitates the pyrimidine de novo pathway flux and elevates pyrimidine synthesis [95] (Figure 4).

### 3.5. Oxidative Metabolism in HCC

**Figure 5:**
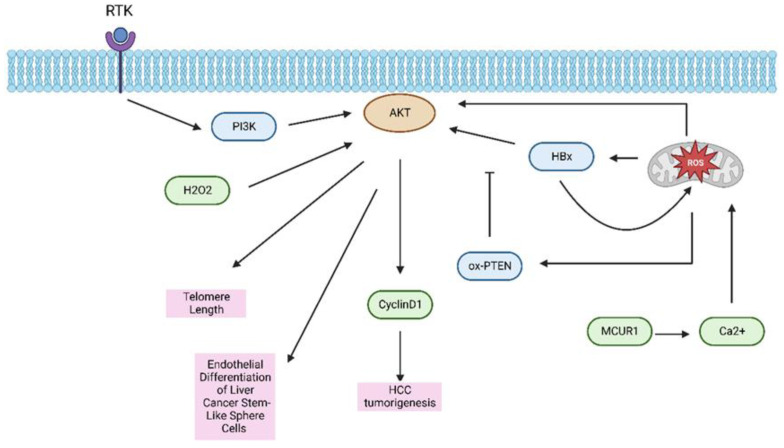
The crosstalk between the PI3K/AKT/mTOR axis and the oxidative metabolism. Abbreviation: HBx, hepatitis B virus x protein; ROS, reactive oxygen species; MCUR1, mitochondrial calcium uniporter regulator 1.

Reactive oxygen species (ROS) are a set of favorably reactive small molecules. The balance of ROS is essential for cell fate, which maintains cell proliferation, differentiation, and promotes cancer metastasis [96]. ROS is positively correlated with DNA damage and the differentiation grade of HCC [97]. Previous research has revealed an interaction between the ROS and AKT signaling pathway [98,99]. Zhao et al. discovered that cancer stem-like sphere cells derived from the human HCC cell line HepG2 may differentiate into endothelial cells by activation of AKT. Thus, targeting the ROS-dependent AKT signal axis may represent a method for human HCC treatment [100]. For the patients with HCC recurrence after liver transplantation (LT), ischemia–reperfusion injury (IRI) is an inescapable outcome after LT, particularly in the early stage, and the investigations have shown that IRI potentially raises the risk of HCC recurrence after LT [101]. Hepatic IRI is intensively related to the presence of ROS in hepatocytes, and a high level of ROS can activate AKT signaling in HCC cell lines [102]. The activation of AKT is caused by ROS production in HCC cell lines, which is consistent with previous observations [103]. The accumulation of ROS is also related to autophagy and apoptosis and impacts the cell cycle regulation of transformed hepatocytes in HCC, as ROS can act as upstream factors to regulate the AKT/mTOR pathway [104]. Similarly, it has been described that the AKT signaling pathway was activated upon ROS generation in the Huh7, HepG2, and BEL7402 human HCC cell lines [105]. In hepatitis B virus (HBV)-related HCC, the HBV plays an important role in HCC formation [106]. The X protein (HBx) coded by the HBV genome is the key regulatory protein in HCC progression [107]. The HBx-induced ROS stimulates the AKT pathway via oxidative inactivation of PTEN, while HBx and ROS preserve a positive regulatory loop, which exacerbates hepatocellular tumorigenesis via cyclin D1 [108]. Furthermore, the ROS-activated AKT is involved in telomere maintenance via H_2_O_2_-mediated AKT activity. Targeting the telomerase activity, which is critical in HCC, by reducing ROS levels through an antioxidant agent or by down-regulating p-AKT levels may become a therapeutic target for HCC patients [109]. Ren et al. have shown that the mitochondrial Ca^2+^ uptake mediated by mitochondrial calcium uniporter regulator 1 (MCUR1) plays a crucial part in the regulation of HCC cell survival by raising ROS production via regulating AKT and p53 [110] (Figure 5). Meanwhile, activation of ROS/AKT signaling has been associated with HCC cell growth and metastasis [111].

## 4. Metabolic Reprogramming in the HCC Tumor Microenvironment

The tumor microenvironment (TME) is a critical contributor to HCC cell growth, invasion, and metastasis [112]. Immune cells, as an essential part of the TME, including expanded infiltrations of T cells and NK cells, are favorable prognostic indicators, emphasizing the prospects of immunotherapy in HCC treatment [113]. Meanwhile, there is growing evidence that the metabolism of cell types in the TME, like immune cells, can modulate tumor progression [114]. Nevertheless, a vicious competition potential exists between tumor cells and immune cells in the tumor microenvironment, and metabolic competition cannot only influence the growth of tumor cells, but can also lead to a loss of immune cell functions due to the lack of nutrition in the microenvironment [115].

Macrophages have been associated with tumor progression and resistance to treatment by delivering malignant cells with nutritional supplements [116]. Lactic acid, created by cancer cells as a product of glycolysis, has an essential function in signaling transduction, contributing to the M2-like polarization of tumor-associated macrophages [117]. In tumors, like HCC, the polarization of the macrophages may be caused by the increased lactic acid concentration in the tumor microenvironment, regulating the macrophage polarization through the AKT pathway [118,119]. However, it has been demonstrated that M1 polarized macrophages have improved glycolytic metabolism and damaged oxidative phosphorylation through the AKT/mTOR/HIF-1α signaling axis [120]. These results underline the importance of the AKT signaling pathway in the M1/M2 polarization of macrophages for HCC.

The consensus opinion of AKT is that it has a universal function in regulating T cell metabolism, but evidence for AKT-independent pathways that regulate T cell metabolism, survival, and proliferation has now been demonstrated [121]. A previous study reported that AKT determines the T cell fates but is not important for regulation of the T cell metabolism [122]. However, the inhibition of the AKT pathway suppresses fatty acid oxidation and enhances the mitochondrial spare respiratory capacity in tumor infiltrating T cells [123].

AKT activation relies on lipid oxidation and short-chain fatty acids instead of glucose or glycolysis in human T regulatory (Treg) cells [124]. The increasing fractions of Treg cells contribute to intrahepatic metastasis of HCC, as Treg cells are one of the immune-inhibition factors that comply with immune repression during the immune effector functions [125]. Programmed death ligand 1(PD-L1) as the immune checkpoint effects the Treg cells by inhibition of the AKT/mTOR/S6 signaling pathway, and enriching PTEN [126]. PD-1/PD-L1 interaction may block mTOR signaling by AKT and PI3K inhibition, thereby decreasing the glycolysis rate of T-infiltrating cells in human HCC [127]. Immunometabolism, which is the metabolic reprogramming after the activation of the immune cells, depends on the crosstalk between PI3K/AKT/mTOR and the LKB1/AMPK (liver kinase B1/AMP-activated protein kinase) signaling pathway, which is critical for regulating both immune and nonimmune cell metabolism [128].

Natural killer (NK) cell-based treatments have been reported as solid and effective therapies for some cancer entities [129]. Activated NK cells undergo significant shifts in cellular metabolic pathways, with a shift towards glycolysis and mitochondrial oxidative phosphorylation (OXPHOS) [130]. Growing evidence suggests that the PI3K–AKT–mTOR pathway is crucial for modulating the development, differentiation, and activation of NK cells [131]. PD-1 may exhibit its inhibitory function on NK cells by decreasing PI3K/AKT signaling in HCC [132]. It was also previously reported that energy metabolism and cell motility deficiencies of NK cells are accountable as prominent mechanisms for NK-cell dysfunction in HCC patients [133]. The metabolic changes in NK cells restrict their effector functions in cancer immune therapy [134].

Due to the metabolic reprogramming, the interaction between PI3K/AKT/mTOR and the metabolism can also alter the immune suppression network, which is widely present in different cancers [135]. The PI3K/AKT/mTOR signaling pathway was shown to be activated in HCC tumor cells with a pronounced glycolytic metabolism leading to the accumulation of lactate.

A dedicated table on the role of metabolism-associated genes, proteins, and molecules from this review can be found in Table 1.

## 5. Targeting the PI3K/AKT/mTOR Pathway for HCC Therapy

The multi-kinase inhibitor regorafenib is used as a second-line agent after sorafenib failure in HCC patients and represents a valuable and relatively safe therapeutic option that brings new hope for HCC therapy [136]. More than half of the HCC patients show constitutive activation of the PI3K/AKT/mTOR pathway [137]. The metabolic dependencies of cancer cells may be further investigated for anti-tumor therapy. For example, various cancer entities are dependent on constitutive signaling through the PI3K/AKT signaling axis. Currently, inhibitors targeting the PI3K/AKT signaling axis and different downstream pathways are in clinical trials [138] (Table 2).

Copanlisib, a PI3K inhibitor recently approved for clinical use, strongly inhibited cell viability and colony formation in HCC cell lines [139]. The study met its primary end point with an objective response rate of 16%, with copanlisib showing promising clinical activity in selected tumors with the *PIK3CA* mutation [140]. Capivasertib has been shown to restrict p53 to the nucleus and activate the autophagy of hepatocytes, indicating that AKT inhibitors may become a potential treatment for HCC patients [141]. Ipatasertib is a highly selective oral small-molecule inhibitor of AKT. In the FAIRLINE randomized clinical trial, a correlation between phosphorylated AKT expression and clinical outcome has been demonstrated in triple-negative breast cancer [142]. Interestingly, the results from this trial implicated that even in the absence of mutations within the *PIK3CA*, *AKT1*, or *PTEN* genes, patients benefitted from ipatasertib treatment.

**Figure 6:**
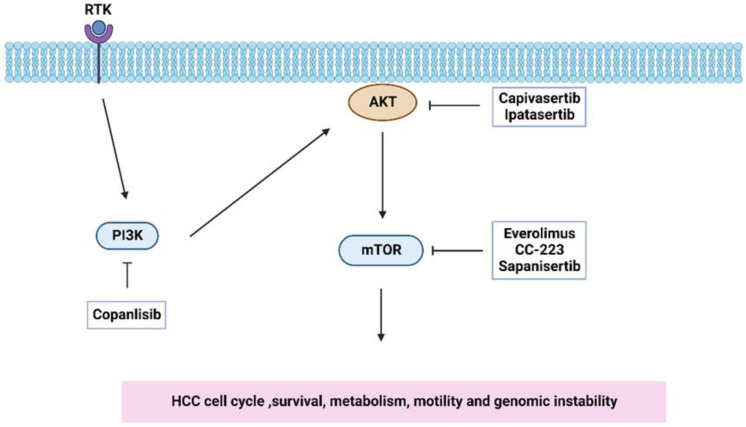
The clinical trial of targeting PI3K/AKT/mTOR pathway on progress based on the Table 2.

Moreover, it has been demonstrated that the mTOR inhibitor everolimus can improve the survival of HCC patients after LT [143]. CC-223, a selective and orally bioavailable mTOR kinase inhibitor, blocked mitochondrial function and conducted ROS production in HCC cell lines [144]. In vitro models as well as murine models mouse model illustrated HCC cell susceptibility for treatment with the mTOR inhibitors rapamycin and sapanisertib [145]. There are some clinical trials targeting this pathway on progress in HCC (Figure 6).

## 6. Present Challenges and Future Directions

Due to the fact that the PI3K/AKT/mTOR pathway is critically involved in several cellular processes, targeting the PI3K/AKT/mTOR pathway leads to adverse events that lead to early treatment or study cancellation. In addition, treatment resistance after PI3K/AKT/mTOR pathway inhibition has been observed. Therefore, new drugs or combination therapies need to be studied to make PI3K/AKT/mTOR inhibitors more tolerable and efficient [146]. Combination therapies are becoming a crucial part of the development of new drug, and a better treatment for cancer [147]. Dual targeting of AKT and mTOR may be a potential treatment option for HCC patients, as demonstrated in preclinical models [148]. Furthermore, clinical trials combining mTOR inhibitors with both chemotherapy and radiotherapy are ongoing [149]. Furthermore, new therapeutic strategies, e.g., radiosensitization, have been proposed for HCC patients. Recently, a new set of selective mTOR inhibitors was developed that increased the radiosensitivity of HCC cells [150]. These results underline the potential applications of PI3K/AKT/mTOR inhibitors as additive treatments or as combination partners for other drugs. The combination of therapies may become the future HCC treatment direction.

## 7. Summary and Conclusions

In recent years, many studies have analyzed the role of the PI3K/AKT/mTOR signaling pathway in the development of HCC [151]. The research on the PI3K/AKT/mTOR signaling pathway contributed to and resulted in the development of inhibitors for HCC treatment. However, the clinical benefits of single-agent therapy using these inhibitors are still limited [152]. The interaction between PI3K/AKT/mTOR signaling and metabolism demonstrates the close connection between the oncogenic signaling network and tumor metabolism. Future research focusing on the crosstalk between the PI3K/AKT/mTOR signaling axis and the cellular metabolism in HCC may help to reveal the impact of metabolic reprogramming in cancer cells and contribute to the development of novel potential therapeutic agents.

## Figures and Tables

**Table 1:** The role of metabolism-associated genes, proteins, and molecules in HCC as discussed in this review.

Gene/Protein/Molecule	Role in HCC	References
4EBP1	Repression of the initiation of protein translation	[45]
6PGD	Key enzyme of the PPP, promoting liver cell growth,	[50,52]
ACC	facilitates the fatty acid synthesis	[63]
AMPK	Regulates growth of HCC CSCs, regulates both immune and nonimmune cell metabolism	[27,128]
ASNS	Asparagine synthetase	[87]
CAD	Pyrimidine metabolism	[92,95]
COX-2	Lipogenesis	[71]
DHODH	Pyrimidine metabolism	[92]
EGFR	Increases proliferation, invasion, and metastasis	[54]
FASN	facilitates fatty acid synthesis	[63]
GDH	Glutamate dehydrogenase	[80]
GLS1	Promotes proliferation	[79]
GLUT1	Glucose metabolism	[44,45]
GS	Glutamine synthetase	[80]
HBx	Regulatory protein in HCC progression, induces ROS	[107,108]
HK2	Glycolysis	[46]
IL-6	HCC development	[38]
Lamc1	Decreases growth of HCC cells	[44]
MCUR1	Regulation of HCC cell survival	[110]
NDFIP1	Initiates metabolic change causing HCC formation and metastasis	[43]
NDRG2	Glutaminolysis	[81]
Nqo1	Glutaminolysis	[86]
NRF2	Triggers transcription of growth factor genes	[73]
PD-L1	Glycolysis rate of T-infiltrating cells	[127]
PHLDA3	Metabolic reprogramming	[53]
ROS	DNA damage and the differentiation grade	[97]
SREBP1	Hepatic cellular lipid metabolism	[64,65,66,67,68]
SREBP2	Hepatic cellular lipid metabolism	[64,65,66,67,68]
SREBPs	Hepatic cellular lipid metabolism	[64,65,66,67,68]
STAT3	Facilitates HCC development	[38]
TIRM21	Metabolic reprogramming	[53]
UBE2T	Pyrimidine metabolism	[92]
UMPS	Pyrimidine metabolism	[92]
VEGFA	Angiogenesis	[24]
VIPR1	Pyrimidine biosynthesis	[94]

**Table 2:** PI3K/AKT/mTOR pathway inhibitors for HCC in clinical trials ^1^.

Inhibitor	Target	Phase	ClinicalTrials.Gov Identifier
Copanlisib	PI3K	2	NCT02465060 Start date: August 2015 Completion date: December 2025
Capivasertib	AKT	2	NCT02465060 Start date: August 2015 Completion date: December 2025
Ipatasertib	AKT	2	NCT02465060 Start date: August 2015 Completion date: December 2025
Everolimus	mTOR	4	NCT02081755 Start date: March 2014 Completion date: January 2023
	mTOR	2	NCT04803318 Start date: January 2021 Completion date: January 2023
CC-223	mTOR	2	NCT03591965 Start date: August 2018 Completion date: December 2022
Sapanisertib	mTOR	2	NCT02465060 Start date: August 2015 Completion date: December 2025

^1^ Clinical trial data was obtained from clinicaltrials.gov in December 2022.

## Data Availability

Not applicable.

## Abbreviations

4EBP1	4E binding protein 1
6PGD	6-phosphogluconate dehydrogenase
ACC	Acetyl-CoA-carboxylase
AMPK	AMP-activated kinase
ASNS	Activation of asparagine synthetase
CAD	Carbamoyl-phosphate synthetase 2, aspartate transcarbamoylase, dihydroorotase
COX-2	Cyclooxygenase 2
CSCs	Cancer stem cells
DHODH	Dihydroorotate dehydrogenase
EGFR	Epidermal Growth Factor Receptor
FASN	Fatty acid synthesis
GDH	Glutamate dehydrogenase
GLS1	Glutaminase 1
GLUT1	Glucose transporter 1
GS	Glutamine synthetase
HBx	hepatitis B x protein
HCC	Hepatocellular carcinoma
HCV	Hepatitis C virus
HK2	Hexokinase 2
IL-6	Interleukin 6
IRI	Ischemia–reperfusion injury
Lamc1	Laminin gamma 1
LT	liver transplantation
MCUR1	Mitochondrial calcium uniporter regulator 1
mTOR	Mammalian target of rapamycin
NAFLD	non-alcoholic fatty liver disease
NDFIP1	Nedd4 family-interacting protein 1
NDRG2	N-Myc downstream regulated gene 2
NK cell	Natural killer cell
Nqo1	NAD(P)H quinone dehydrogenase 1
NRF2	Nuclear factor erythroid 2-related factor 2
OXPHOS	Oxidative phosphorylation
PDGFR	Platelet-derived growth factor receptors
PD-L1	Programmed death ligand 1
PHLDA3	Pleckstrin Homology Like Domain Family A Member 3
PI3K	Phosphatidylinositol 3-Kinase
PKB	Protein kinase B
PLC	Primary liver cancer
PPP	Pentose phosphate pathway
PTEN	Phosphatase and tensin homolog
ROS	Reactive oxygen species
S6K1	Protein S6 kinase 1
SIRT4	Sirtuin 4
SREBP1	Sterol regulatory-element binding protein 1
SREBP2	Sterol regulatory-element binding protein 2
SREBPs	Sterol regulatory-element binding proteins
STAT3	Signal transducer and activator of transcription 3
TCA	Tricarboxylic acid
TIRM21	Tripartite motif-containing protein 21
TME	Tumor microenvironment
Treg cell	T regulatory cell
UBE2T	Ubiquitin-conjugating enzyme E2T
UMPS	Uridine 5′-monophosphate synthase
VEGFA	Vascular endothelial growth factor A
VIPR1	Vasoactive intestinal polypeptide type-I receptor
